# COVID-19 and Laboratory Markers from Romanian Patients—A Narrative Review

**DOI:** 10.3390/life13091837

**Published:** 2023-08-30

**Authors:** Ovidiu Musat, Virgiliu Bogdan Sorop, Madalina Ioana Sorop, Viorica Lazar, Daniela Teodora Marti, Monica Susan, Cecilia Roberta Avram, Andrada Oprisoni, Dan Dumitru Vulcanescu, Florin George Horhat, Iulia Cristina Bagiu, Delia Ioana Horhat, Mircea Mihai Diaconu

**Affiliations:** 1 Department of Ophthalmology, “Carol Davila” University of Medicine and Pharmacy, Dionisie Lupu Street, No. 37, Sector 2, 020021 Bucharest, Romania; ovidiumusat@yahoo.com; 2 Department of Ophthalmology, “Dr Carol Davila” Central Military Emergency University Hospital, Mircea Vulcanescu Street, No. 88, 010825 Bucharest, Romania; 3 Department of Obstetrics and Gynecology, “Victor Babes” University of Medicine and Pharmacy, Eftimie Murgu Square, No. 2, 300041 Timisoara, Romania; bogdan.sorop@gmail.com (V.B.S.); diaconu.mircea@umft.ro (M.M.D.); 4 Doctoral School, “Victor Babes” University of Medicine and Pharmacy, 300041 Timisoara, Romania; pop_madalina_91@yahoo.ro (M.I.S.); dan.vulcanescu@umft.ro (D.D.V.); 5 Department of General Medicine, “Vasile Goldis” University of Medicine, Liviu Rebreanu Street, No. 86, 310048 Arad, Romania; lazar.viorica@uvvg.ro; 6 Pediatric Clinic II, Clinical Hospital Emergency of Arad County, Andrényi Károly Street, No. 2-4, 310037 Arad, Romania; 7 Department of Biology and Life Sciences, “Vasile Goldis” University of Medicine, Liviu Rebreanu Street, No. 86, 310048 Arad, Romania; dana_m73@yahoo.com; 8 Clinical Analysis Laboratory Clinical Hospital Emergency of Arad County, Andrényi Károly Street, No. 2-4, 310037 Arad, Romania; 9 Department of Internal Medicine, Centre for Preventive Medicine, “Victor Babes” University of Medicine and Pharmacy, Eftimie Murgu Square, No. 2, 300041 Timisoara, Romania; monisusan@yahoo.com; 10 Department of Residential Training and Post-University Courses, “Vasile Goldis” Western University, Liviu Rebreanu Street 86, 310414 Arad, Romania; avram.cecilia@uvvg.ro; 11 Department of Pediatrics, Discipline of Pediatric Oncology and Hematology, “Victor Babes” University of Medicine and Pharmacy, Eftimie Murgu Square, No. 2, 300041 Timisoara, Romania; oprisoni.licinia@umft.ro; 12 Department of Microbiology, “Victor Babes” University of Medicine and Pharmacy, Eftimie Murgu Square, No. 2, 300041 Timisoara, Romania; horhat.florin@umft.ro; 13 Multidisciplinary Research Center on Antimicrobial Resistance (MULTI-REZ), Microbiology Department, “Victor Babes” University of Medicine and Pharmacy, Eftimie Murgu Square, No. 2, 300041 Timisoara, Romania; 14 Clinical Analysis Laboratory, “Louis Turcanu” Emergency Clinical Hospital for Children, Iosif Nemoianu Street 2, 300011 Timisoara, Romania; 15 Department of ENT, “Victor Babes” University of Medicine and Pharmacy, Eftimie Murgu Square, No. 2, 300041 Timisoara, Romania; horhat.ioana@umft.ro

**Keywords:** COVID-19, SARS-CoV-2, laboratory markers, biomarkers, diagnostic

## Abstract

COVID-19 has significantly impacted the whole world, and Romania was no exception. Biomarkers play a crucial role in understanding and managing the disease. However, research regarding laboratory analyses for patients with COVID-19 is fairly limited. For detection, PCR testing is still considered the golden standard, while antibodies are still useful for monitoring both patients and their vaccination status. In our country, biomarkers such as CRP, LDH, transaminases, cardiac, and iron markers have been used to assess the status of patients and even predict illness outcome. CRP, IL-6, LDH, FER, fibrinogen, creatinine, and vitamin D levels have been associated with increased severity, risk of ICU admission, and death. Cardiac markers and D-dimers are also good predictors, but their role seems more important in patients with complications. HDL cholesterol and BUN levels were also suggested as potential biomarkers. Hematological issues in SARS-CoV-2 infections include neutrophilia, lymphopenia and their ratio, while PCT, which is a marker of bacterial infections, is better to be used in patients with co- or supra-infections. The current research is a narrative review that focuses on the laboratory results of Romanian COVID-19 patients. The goal of this article is to provide an update on the research on biomarkers and other laboratory tests conducted inside the borders of Romania and identify gaps in this regard. Secondly, options for further research are discussed and encouraged.

## 1. Introduction

### 1.1. Background Information on COVID-19 and Its Impact in Romania

COVID-19, caused by the severe acute respiratory syndrome coronavirus 2 (SARS-CoV-2), emerged in late 2019 and quickly spread across the globe. The World Health Organization (WHO) declared it a pandemic in March 2020, highlighting the urgent need for countries to implement measures to control the spread of the virus [1].

One of the most concerning aspects of COVID-19 is its high level of contagiousness. The potential to transmit the virus begins prior to the development of symptoms and is highest in the first few days after symptom onset [2]. Several comorbidities that increase the risk of severe illness and death from COVID-19, including heart disease, diabetes, and obesity, have been previously described [3]. Although initially there were few pediatric cases, children have been observed to be able to also be infected with the virus and transmit it to others [2].

With its highly contagious nature, the virus posed significant challenges to healthcare systems worldwide. The COVID-19 pandemic has had a profound impact on countries around the world, and Romania is no exception. As the virus began to spread globally, Romania faced the challenge of managing the outbreak within its borders [1]. The Romanian government swiftly implemented measures to contain the virus and protect its population, including travel restrictions, lockdowns, and the closure of non-essential businesses. Testing and contact tracing efforts were also ramped up to identify and isolate individuals who had been in close contact with confirmed cases [4].

Since the start of the pandemic, Romania has experienced several waves of COVID-19 infections. The country has seen fluctuating numbers of daily new cases, with peaks and troughs in transmission rates. The virus has resulted in a significant number of hospitalizations and deaths. Mortality rates have varied over time, with spikes during periods of high transmission. The impact of the virus on vulnerable populations, such as males, the elderly, and those with pre-existing health conditions (hypertension, diabetes, obesity, chronic kidney disease, and diseases of the circulatory system), has been particularly severe [3].

According to a study implementing the Fear of COVID-19 Scale (FCV-19S), a valid and reliable tool for assessing fear of COVID-19 among Romanian adults, the research highlighted the inverse relationship between resilience, happiness, and fear and discussed the particularities of the global health crisis in Romania [5]. The pandemic has had a huge effect on the psychosocial behavior of Romanians [6,7].

Vaccination campaigns started in Romania at the end of 2020. However, the country has recorded lower vaccination rates compared to neighboring EU countries. Vaccine hesitancy has been identified as a major factor contributing to low vaccine uptake. Increasing vaccination coverage and addressing vaccine hesitancy are crucial for reducing the spread of the virus [4,8].

Focusing on patients from Romania, particularly in the context of COVID-19, can be important for several reasons. Generally, understanding and addressing the impact of COVID-19 in Romania can contribute to the overall public health response, provide insight into virus spread, treatment effectiveness, strain on the healthcare system, and assess the impact of the virus on vulnerable populations, such as the elderly (as Romania has a generally aged population) or Roma patients.

### 1.2. Importance of Laboratory Findings in Understanding and Managing the Disease and Purpose of the Study

**Figure 1:**
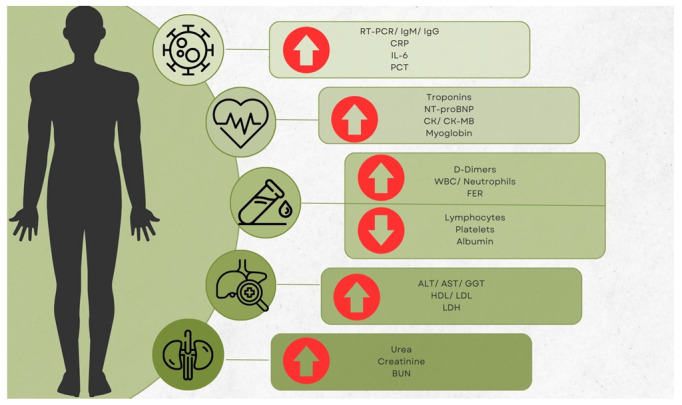
Main laboratory modifications noticed on an international scale.

Biomarkers have long been crucial in the clinical decision-making process for a variety of infectious illnesses. In order to determine the severity of a disease and the proper distribution of resources, it is imperative to evaluate the function that biomarkers play. The identification of high-risk patients and the proper allocation of healthcare resources during the pandemic may be carried out with the use of laboratory data, which have the potential to be utilized as early indicators to enhance the management of COVID-19 patients. Numerous proinflammatory cytokines, including c-reactive protein (CRP), lactate dehydrogenase (LDH), aspartate transaminase (AST), neutrophil count, neutrophils-to-lymphocytes ratio (NLR), troponins (Tn’s), creatine kinase (CK), D-dimers, and brain natriuretic peptide (BNP), are among the most often utilized COVID-19 indicators (Figure 1) [2,9,10,11,12].

The present paper is a narrative review focusing on laboratory findings in COVID-19 patients from Romania. The objective of the present article is to provide an update regarding the study of biomarkers and other laboratory analyses performed within the Romanian borders. In addition, this work attempts to identify gaps in research coming from this country and emphasizes the need to tackle these unique features to contribute to the worldwide knowledge of the pandemic. As such, a secondary objective is to provide encouragement to Romanian authors to work on novel and detailed aspects of COVID-19. Arrow up means elevated; Arrow down means decreased. 

## 2. Materials and Methods

### 2.1. Study Design, Sample Selection and Data Collection

The present paper is a narrative review focusing on laboratory findings in COVID-19 patients from Romania. The research was conducted on 2 electronic databases (PubMed and Google Scholar) from 1 May 2023 to 1 July 2023. Consulting a medical librarian, the following advanced search was performed: (((COVID-19) OR (SARS-CoV-2)) AND (Romania) AND ((laboratory) OR (biomarker) OR (paraclinical)) NOT (review)).

A total of 626 records were identified through primary screening conducted by multiple examinators (OM, VBS, MIS, and DDV). The records were further evaluated, and duplicates were removed (n = 258). The remaining 368 papers were read fully, and the ones that did not focus on any of the studied aspects were removed, resulting in 89 final original articles containing data from Romanian patients. Pertinent review articles were also screened for supplementary and complementary references that might have been missed in the primary search. As such, the inclusion criteria were original articles featuring patients and laboratory data from Romania, articles in Romanian or English, and articles featuring pediatric and/or adult patients. Exclusion criteria were as follows: articles featuring patients and data from Romania pooled together with other nationalities; articles that were reviews, systematic reviews, metanalyses, or correspondence articles such as letters to the editor; articles with incomplete data on laboratory findings. In order to reach a consensus and reduce the possibility of bias and misinterpretation, the group participated in a follow-up meeting that addressed data validation and solved any major disagreements. Articles were also excluded if they provided insufficient data or presented methodological flaws.

### 2.2. Limitations

As with all narrative reviews, the present paper is not exempt from limitations. Bias risks are inherent to these types of articles, which could be reduced by the writing of a systematic review or a meta-analysis. Non-randomized and observational studies also possess inherent limitations and biases. Additionally, data can be presented heterogeneously, with some articles featuring normally distributed data (mean and standard deviation) and some featuring non-parametric data (median and IQR). Lastly, the study is aimed only at data from Romanian patients, which could not be representative of other countries.

## 3. Results

### 3.1. PCR Testing

As previously explored, PCR testing was the most commonly used method of detecting active cases of COVID-19 and can even be considered the gold standard for doing so [12]. Among the first to study the utility of PCR testing in Romania, the study by Petrovan et al. aimed to compare commercial kits that are used in COVID-19 diagnostics in terms of their sensitivity and feasibility for use in pooling. It showed that pooling of up to 80 samples did not affect the efficacy of the kits if the initial positive sample had a high viral load (Cq > 16) and that the RNA-dependent RNA polymerase (RdRp) gene is a more suitable target in pooled samples than the envelope (E) gene, which could provide an easier method of screening a large number of samples [13]. The following year, in 2021, the same topic was discussed by Cruceriu et al., with their findings focusing on the fact that the pooling method can reduce the cost and number of tests needed for large-scale testing while maintaining a high level of accuracy. Furthermore, they stated that this method provides a multifunctional approach, as the pooling of samples can be carried out in a variety of ways, including by sample type, by geographic region, or by laboratory [10].

In 2022, a study by Motoc et al. covered the number of days necessary for patients with mild and moderate forms of COVID-19 to reach undetectable levels of SARS-CoV-2 RNA in the upper respiratory tract specimens. The number of days with a traceable viral load was 25.93 (±6.02) days in patients with mild COVID-19 and 26.97 (±8.30) days in patients with moderate COVID-19. Age, male gender, and obesity, along with several chronic conditions (cardiac, liver, renal, and neurological disease), were associated with prolonged positive RT-PCR tests from the nasal swab (therefore prolonged viral load) [14].

Considering that for the PCR test, the nasal and pharyngeal mucosa must be swabbed, the study by Cismaru et al. followed how this method of sample collection can affect the patient at that specific moment. A COVID-19 aerosol-generating event (AGE) refers to a situation or activity that has the potential to generate respiratory aerosols, which are tiny particles containing the SARS-CoV-2 virus that causes COVID-19. The prevalence of AGEs triggered by the nasopharyngeal swabbing in the study was 21.23%. No differences were observed between positive and negative groups; however, male participants showed a higher AGE occurrence [15].

Vertical transmission from pregnant mothers to their children of SARS-CoV-2 is still a contested issue even to this day [16]. Still, in the study by Citu et al. from 2021 addressing this topic, the placenta was the most common area for SARS-CoV-2 analysis to be found positive, being observed in 7.53% of the tests. They also observed that breast milk was found to be a safe feeding source, with just 1.01% of positive PCR test results [17].

### 3.2. C-Reactive Protein (CRP)

CRP is a protein made by the liver that acts as an early indicator of inflammation and infection. The normal level of CRP in the blood is less than 5 mg/L, but it rises quickly within 6 to 8 h and reaches its greatest peak 48 h after the commencement of the disease. Its half-life is approximately 19 h and its concentration diminishes as the patient heals and the inflammatory stages pass [18,19]. This particular protein was studied extensively during the COVID-19 pandemic, as it is one of the earliest markers to show elevation since the beginning [14,20,21,22,23,24,25,26,27,28,29,30,31,32,33,34,35,36,37,38,39,40,41].

CRP can also be observed in other conditions, such as urticaria exacerbations or other cutaneous manifestations, where it has proven to have a significant association [42,43]. Another such correlation was observed by Cerbu et al. in their study regarding patients with hepatitis, where, due to the liver impairment caused by the underlying condition, statistically significant differences were observed between patients with hepatitis and those without (56 mg/L vs. 12 mg/L) [44]. Other conditions correlated with CRP levels in SARS-CoV-2 infection are heart conditions according to Pilut et al. and Tudoran et al. [33,45,46], thromboembolic events due to lupus antibodies, as observed in a multivariate analysis by Dima et al. (OR = 1.008, 95% CI: 1.001–1.016) [47], and type 2 diabetes according to Restea et al. and Gradisteanu et al. [48,49]. Another study from 2022 investigated a link between laboratory markers and features found on the Computer Tomography (CT) results. The findings demonstrated a significant relationship between high CRP levels and CT severity features in COVID-19 patients at an R value of 0.40 [50]. In addition, an interesting finding was reported by Bende et al. in 2021, assessing those higher levels of CRP as being associated with the occurrence of cardiac abnormalities but not with liver abnormalities [51]. The study by Miftode et al., which focused on heart failure patients positive for COVID-19, found that CRP levels were higher in patients from rural areas and with better levels of finance, with which a weak correlation was established [52].

Regarding the use of CRP in specific populations, studies by Mocanu et al. observed that elevated levels were statistically higher in Roma patients versus the general population (almost twice as elevated); however, they could not be considered predictive markers for ICU admission and death in this specific group [53,54]. Regarding vaccinated patients, the study by Pavel-Tanasa indicated no significant variations based on gender, with significantly lower amounts in the serum of previously infected individuals compared to infection-naive individuals [54,55].

There were three studies focusing on pediatric patients. Firstly, in Totan et al.’s study, CRP levels were associated with increased cases of abdominal pain, nausea, and vomiting [56]. In Bagiu et al.’s study, which compared infants (<1 year) to older children, median values were statistically higher in the older children’s group, which promotes the idea that it performs well in its role as a biomarker in these patients with COVID [57]. Another article, by Capraru et al., performed a more in-depth study, first by comparing how several biomarkers affect children, adults, and elders, and second by calculating cutoff values for these biomarkers according to age. In regard to the possibility of ICU admission, a cutoff value of 22.51 was calculated (sensitivity (Se): 85.71% and specificity (Sp): 84.74%), while in regards to mortality, the cutoff value was 13.42 at 100% sensitivity and 62.96% specificity [58].

Regarding adults and elders and the risk of ICU admission and death, both Capraru et al. and Pal et al. observed that elevated levels can be used as a predictive marker; however, the cutoff values were heterogenous [58,59]. Similarly, Timpau and his team described CRP as a good, independent ICU admission and mortality risk factor [60]. Furthermore, Paranga et al. concluded that the best role for CRP as a biomarker was to distinguish between severe and non-severe cases [61]. CRP levels were also associated with new neurological manifestations in elder patients [62] and children with pediatric inflammatory multisystem syndrome (PIMS) [41,63]. In the rare cases of vertical transmission, such as the one identified by Iacob and his team, CRP was elevated in the newborns with positive SARS-CoV-2 PCR testing [64].

A study by Marc et al. followed how this marker changed due to treatment use. At the time, the traditional course of treatment, as recommended by the Romanian Ministry of Health, entailed symptomatic medications such as acetaminophen for fever, codeine phosphate or acetylcysteine for cough, metoclopramide for vomiting, anticoagulants such as warfarin, corticosteroids such as dexamethasone, which has anti-inflammatory effects, as well as antivirals such as Favipiravir or Remdesivir [65,66]. In the end, the authors decided to use Tocilizumab and Anakinra, both in association with Remdesivir, in patients who had developed a cytokine storm due to COVID-19 pneumonia. Initially, CRP levels were similar (132 mg/dL for Tocilizumab and 130.3 mg/dL for Anakinra), but a difference in value dynamics was observed starting with day 4 (85 vs. 108 mg/dL), which continued until day 10 (24.6 vs. 40 mg/dL) [67].

Another study conducted by Novacescu and his team, with a focus on using therapeutic plasma exchange followed by convalescent plasma transfusion in severe and critically ill COVID-19 patients, observed a significant decrease in CRP in the treatment group with an increase in survival days [68]. Another study with a focus on convalescent plasma treatment found similar modifications [69].

In regard to treatment and CRP values, it was observed that patients with CRP levels greater than 75 mg/L were more likely to have severe disease and benefit more from Tocilizumab treatment [70].

### 3.3. Cytokines and Other Proinflammatory Markers

In the body, proinflammatory cytokines can be activated and released by SARS-CoV-2. Cytokine markers are a class of polypeptide signaling molecules that may activate and control a wide range of cellular biological activities. Some of the most important cytokines are interleukins, including IL-1, IL-4, IL-6, IL-7, IL-10, IL-12, IL-17, and IL-18, as well as gamma-interferon (IFN-γ), tumor necrosis factor alpha (TNF-α), transforming growth factor beta (TGF-β), and nuclear factor kappa-light-chain-enhancer of activated B cells (NF-kB), have been found to play significant roles in the body’s inflammatory response to SARS-CoV-2 infection [71].

In Romanian patients, increased levels of IL-1 and IL-6, of which the second was by far the most reported, have been reported on numerous occasions in both adults and children [25,27,30,33,36,40,43,72]. Timpau et al. observed that IL-6 was strongly associated with ICU admission {OR = 2.62) [60]. Tudoran et al. reported that the β coefficient for IL-6 was positive (0.526), indicating that higher levels of IL-6 were associated with higher levels of estimated systolic pulmonary artery pressure and altered left ventricular systolic function [45,73]. The study by Pal et al. advocated for IL-6 as a possible marker for mortality (at a cutoff of 27.68 pg/mL) [59]. The study by Mocanu et al., which focused on comparing the laboratory markers of patients from the Roma groups, found that this population had higher levels of IL-6 when compared to the general population [53].

There were some studies that reported no changes in IL-6 levels [21,74]. Radulescu et al. observed that repeated IL-6 measurements were not relevant for outcome prediction because the study observed a significant increase by days 3 and 7 in most patients. Therefore, they concluded that CRP was a more reliable marker in dynamics than IL-6 [70].

In regard to therapeutic approaches, the transfusion of convalescent plasma led to a decrease in IL-6 levels [69]. The study by Pilut et al. from 2022 also observed a difference in severity between groups in regard to TNF-α, while the study by Mocanu et al. could not establish such a relationship [33,53].

A study by Hutanu et al. from 2022, which aimed to evaluate the changes in total natural killer (NK) cells and different NK subpopulations in COVID-19 patients, found four distinct NK sub-populations, based on the differentiated expression of surface markers. There was no difference between the total NK percentage of different disease forms, but the total numbers decreased significantly both in survivors and non-survivors. The main cytokine producers gradually declined during the study period in the survival group, underscoring the importance of adequate IFN production during the early stages of SARS-CoV-2 infection. Persistency in the circulation of CD56 ++ NK cells may have prognostic value in patients with a fatal outcome [75].

The study conducted by the team consisting of Trofin and her colleagues aimed to research the levels of cytokines and chemokines in breastmilk of mothers who had been infected with or vaccinated against COVID-19. Overall, the presence of these cytokines and chemokines in breastmilk may help to provide some protection against COVID-19 for the breastfed infant. The paper observed that TNF-α, IL-6, IFN-β, IL-10, IL-1β, IFN-γ, IL-2, GM-CSF, IL-5, and IP-10 were all found in breastmilk [76]. IL-6 and IL-1β were also associated with severity in pediatric patients with diabetes, as presented by Restea et al. [48]. A further study on interleukins was conducted by Gradisteanu et al., and they observed that individuals with type 2 diabetes who were infected with SARS-CoV-2 had elevated levels of IL-8 and tended to have higher levels of IL-1β and IL-17 compared to healthy controls. They also found an association between IL-8 and IL-17 levels and specific bacteria in these patients, while IL-1β, IL-8, IL-17, and CRP were positively linked to a higher abundance of fungi [49].

### 3.4. Lactate Dehydrogenase (LDH)

LDH, an intracellular enzyme involved in energy generation, is one possible biomarker whose increased blood levels may signal the severity of COVID-19 [77]. In cases of tissue damage and consequent cell death, hypoxia (occurring during respiratory failure), disorders of the hematological and lymphatic systems, or inflammation of the lungs, pericardium, and pancreas, an elevated quantity of this enzyme in the blood was seen. The heart, lungs, liver, and skeletal muscle have the largest quantities of these substances [78]. An increase in LDH activity was seen in many cases of severe COVID-19, which may be related to cell destruction as well as poor blood flow and oxygen delivery. Elevated LDH levels were observed in many studies of Romanian patients [21,26,33,34,79], with one study focusing on how these values could be related to thrombotic microangiopathy by presenting a case report of a patient with a complement gene variant (complement factor I) [80] and another focusing on a pediatric case, signaling the risk for a systemic inflammatory syndrome associated with COVID-19 in some children [23]. Neonatal vertical infection is rare; however, in those cases, LDH was elevated [64].

The research of Bagiu et al. and Capraru et al. also focused on LDH, with the observation that no statistically significant differences were observed between infants (<1 year) and older children; however, it was associated with increased severity and mortality in adults, elders, and especially children (the LDH area under the curve (AUC) of children was bigger than both that of adults and elders at the following values: 0.936 vs. 0.729 and 0.740, respectively). The following cutoff values for the risk of ICU admission were calculated: >302 in adults (Se. = 78.69%, Sp. = 74.53%), >288 in children (Se. = 80%, Sp. = 67.74%), and >184 for elders (Se. = 78.35%, Sp. = 56.48%), while for the risk of death, the values were >294 in adults (Se. = 74.47%, Sp. = 68.1%), >354 in children (Se. = 100%, Sp. = 87.12%), and >195 for elders (Se. = 85.45%, Sp. = 55.33%) [57,58]. The use of LDH as a biomarker in the context of SARS-CoV-2 infections was also supported by the study by Paranga et al., with similar cutoff values [61], concluding that it can be used as an independent prognostic factor.

The study by Marc et al. shows that the comparative evolution of LDH in the two groups (tocilizumab and anakinra) shows an almost equal initial value (475.6 vs. 475.4 u/L) on days three to four; a more pronounced decrease is observed in the tocilizumab group (327.2 vs. 403.2 u/L), and on the tenth day, the LDH value is lower in the tocilizumab group, reaching normal values (221.1 vs. 252.8 u/L in the anakinra group) [67]. In the study by Novacescu and his team, with a focus on using therapeutic plasma exchange followed by convalescent plasma transfusion in severe and critically ill COVID-19 patients, they observed a significant decrease in LDH in the treatment group, with an increase in survival days [68]. In the study by Radulescu et al., it was found that Tocilizumab reduced the proinflammatory state with a sharp decrease in LDH from baseline by day 7 [70].

### 3.5. Creatine-Kinase (CK) and Its Isoforms

CK levels are usually observed in the case of muscle lesions. In many infections, especially viral, this biomarker may increase due to the rhabdomyolysis these microorganisms exhibit during the course of the infection. Although not lethal, this symptom is unpleasant and has been observed infrequently in COVID-19 cases. The isoform CK-MB, associated mostly with cardiac muscle tissue, seems to be somewhat more specific [81].

As such, a few studies that observed increased levels of CK and CK-MB include Draganescu et al. from 2021, Cocos et al. from 2022, and Pilut et al. from 2022 [21,26,33].

Of note is the study by Zaharie et al., which focused on newborns from SARS-CoV-2-positive mothers, where some infants had elevated CK values that returned to normal after three days. The PCR tests for the newborns were negative [82].

There were also studies that did not find any significant changes in CK levels. This also applied to some pediatric cases [57]. Speaking of which, the study by Melit et al. observed CK-MB to be more frequently associated with PIMS than with SARS-CoV-2 infection [83].

### 3.6. Troponins (TN’S) and Other Cardiac Markers

Considering that the main gateway inside the cells is the angiotensin-converting enzyme 2 (ACE2), which is abundant in the cardiac tissue, many authors have suggested a deep inspection of cardiac markers, the most important being the troponins (especially isoform I) and the brain natriuretic peptide (NT-proBNP). Myoglobin was initially proposed as well; however, it was found to be too non-specific [84,85].

Normal cardiac markers were observed in a few studies [21,86], while elevated levels were observed in others [23,37,45,63,74]. Furthermore, cardiac markers varied by NYHA classification and were considered an independent risk factor [33]. Regarding NT-proBNP, Melit et al. in 2022 revealed that patients with PIMS had higher chances of presenting significantly increased levels (OR = 4.111, 95% CI: 1.315–12.854), data that were corroborated by Mihai et al. in 2023 [41,83].

### 3.7. Ferritin (FER) and Other Iron Markers

Anemia of inflammation, hypoferremia, poor transferrin saturation, and high levels of serum ferritin, hepcidin, lipocalin-2, catalytic iron, and soluble transferrin receptor (in ICU patients) have all been iron-associated changes in COVID-19 from the beginning [87].

Low levels of iron have been reported in Romanian patients early on, accordingly to international data [56,72,88]; however, there have been cases of normal values reported [54,86,89,90], while others have reported high levels of FER, including in children [14,21,23,24,25,26,29,30,31,35,36,41,43,48,62,74,79], with a few rare cases of vertical transmission describing increased FER levels, as observed in Iacob et al.’s study [64].

While studies such as the ones conducted by Timpau et al. and Radulescu et al. found an association between FER levels and ICU admission [60,70], the detailed risk for ICU admission and the role of FER were assessed by Capraru et al. at the beginning of 2023. Their discoveries are as follows: for adults the cutoff point was 529 (Se. = 60.78%, Sp. = 89.35%), for elders it was 133 (Se. = 80.85%, Sp. = 60.4%), and for children it was 211 (Se. = 100%, Sp. = 86.02%). Further, they also calculated the risk of death, which returned the following values: for adults, 529 (Se. = 88.57%, Sp. = 89.83%), for elders, 155 (Se. = 80%, Sp. = 52.86%), and for children, 415 (Se. = 100%, Sp. = 98.95%) [58].

It was observed that FER did not decrease significantly within 7 days, neither in survivors nor in non-survivors, after Tocilizumab administration, as it depends more on IL-18. As such, it is not recommended to use it as a marker in Tocilizumab use [70].

In pregnant women, other iron markers, such as sideremia and transferrin, were reduced, and haptoglobin was increased. In addition to the modifications of all iron markers in this study, reticulocyte counts were also low. In regard to correlations, this article also observed a relationship between the low levels of iron in COVID-19-positive patients and the risk of emergency c-section and prematurity. The authors suggested, therefore, the supplementation of iron [88]. Contrary to this, the study by Pinte et al. described a decrease in haptoglobin and schistocytes on the peripheral blood smear [80].

Admission hepcidin, another iron-regulating molecule dependent on IL-6, was significantly higher in patients with multiple organ dysfunction (MOD) (328.7 pg/mL vs. 194.1 pg/mL) and patients with anemia in the context of COVID-19. In the end, it did not prove efficient at predicting the occurrence of death [74]. However, no changes were observed in the study by Szabo et al. when it came to hepcidin and its role in Tocilizumab treatment [90].

### 3.8. High- and Low-Density Lipoprotein Cholesterol Markers (HDL and LDL), Triglycerides

It is now known that cholesterol is a chemical that controls how the SARS-CoV-2 virus enters the host cell. The relationship between lipid-associated diseases and COVID-19 illness is still up for dispute, and there is a lack of information about the potential involvement of cholesterol-carrying lipoproteins and their receptors in relation to infection. As a virus scavenger, immunological modulator, and mediator of viral entry, HDL appears to play a range of roles. While the lipid metabolic pathways and the composition of membranes might be addressed to specifically disrupt the life cycle of the virus as a foundation for antiviral therapy, cholesterol and lipoproteins are promising indicators for monitoring the viral infection state. Higher levels of LDL-C and HDL-C were negatively linked with CRP. Although a fairly regular test, triglyceride levels were not consistently found to be significantly associated with an increased risk [91,92].

In 2023, Capraru et al. focused on studying the associations between HDL levels and the risk of ICU admission and mortality. The following cutoff values for ICU admission were established: 32.81 for adults (Se. = 71.6%, Sp. = 59.31%), 47.75 for children (Se. = 85.71%, Sp. = 56.11%), and 33.17 for elders (Se. = 48.91%, Sp. = 80.58%). For the association with risk of death, the values were 32.03 for adults (Se. = 69.23%, Sp. = 60.04%), 41.35 for children (Se. = 100%, Sp. = 67.4%), and 30.99% for elders (39.13%, Sp. = 81.21%) [58].

The findings of Gradisteanu et al. suggest that individuals with type 2 diabetes may have a higher risk of developing cardiovascular disease in the context of COVID-19 due to lower HDL and higher LDL levels when compared to a non-diabetic control with SARS-CoV-2 infection [49]. The works of Tudoran et al. from 2023 report the correlation between HDL and LDL levels and various cardiovascular parameters, such as aortic and arterial stiffness and diastolic dysfunction. The results suggest that abnormal HDL and LDL levels may be associated with increased cardiovascular risk in patients recovering from COVID-19 [46,93]. Some triglyceride involvement was observed in patients known to have metabolic syndrome [46,93,94].

### 3.9. Transaminase Levels and Other Hepatic Markers

Some of the earliest proposed biomarkers were aspartate aminotransferase (AST) and alanine aminotransferase (ALT) levels, which are markers of hepatic cell lysis [95]. In the beginning, these levels were elevated in some patients (around one quarter, as described by Wijarnpreecha et al. in their 2021 meta-analysis) [96]. Later, it turned out that the power of these analyses as biomarkers for COVID-19 severity was and is disputed. In Romania, one of the first authors to observe that the differences between patients with severe and non-severe cases were not statistically significant was Totan et al. [56]. Other studies had similar findings [28,32,60].

On the other hand, studies such as those by Bende et al. and Laza et al. observed a significant difference in both ALT and AST levels, when comparing COVID-19-positive patients with and without pulmonary injuries, suggesting a link to disease severity affecting the lungs [43,51]. The study by Davidescu et al. observed a statistically significant difference between elders and non-elders in regard to AST [62]. Other cases were reported with elevated levels as well, with a tendency towards elevated AST rather than a modified ALT [23,24,25,26,30,34,35,36,37,59,64,79,97].

In some cases, modified transaminase levels can linger or appear tardively, leading to some cases being diagnosed as post-COVID immune hepatitis [21]. Although considered generally safe for use, Remdesivir was used in pediatric patients as treatment, and it was observed that one important side effect is the elevation of both AST and ALT [98].

Bilirubin is a marker which has also been researched. Only the study by Melit et al. could establish a link between this marker’s decreased levels and PIMS [83].

### 3.10. Vitamin D (25-OHD)

Vitamin D is a pluripotent hormone that has important connections to the immune response. It stimulates the generation of anti-inflammatory cytokines and produces cathelicidins and defensins, which can slow virus multiplication. In addition, Vitamin D inhibits hyperinflammatory responses while also hastening the healing process in the injured regions, particularly in lung tissue [99,100].

More cardiovascular, neurological, and pulmonary conditions, as well as diabetes and cancer, were prevalent in vitamin-D-deficient people. Vitamin-D-deficient individuals exhibited significantly greater risks of severe/critical forms of COVID-19 [OR = 1.23 (95% CI 1.03–1.47)] and mortality [OR = 1.49 (95% CI 1.06–2.08)] in multivariate logistic regression models. In hospitalized COVID-19 patients, vitamin D insufficiency was linked to illness severity and mortality [101].

Lower levels of vitamin D have been associated with severity, which in turn represents an inverse relationship with other markers such as CRP, D-dimers, or Il-6 [102]. This also seems to be applicable to children [57]. The study by Capraru et al. followed up on this idea and established cutoff points in regards to ICU admission—20.5 for adults (Se. = 83.05%, Sp. = 73.71%), 17.07 for children (Se. = 81.82%, Sp. = 76.35%), 22.44 for elders (Se. = 86.81%, Sp. = 35%), and death—17.34 for adults (Se. = 85%, Sp. = 84.74%), 14.11 for children (Se. = 100%, Sp. = 78.98%), and 17.88 for elders (Se. = 54%, Sp. = 67.38%) [58].

Although differences were observed due to disease severity, the study by Hutanu et al. did not manage to find significant differences between low vitamin D patients and ones with normal values regarding markers such as CRP, blood counts, FER, or fibrinogen [29]. Furthermore, the study by Pavel-Tanasa et al. contested the role of vitamin D in the context of BNT162b2 vaccination [55].

### 3.11. Procalcitonin (PCT)

Procalcitonin is a protein that, in normal circumstances, is produced in response to bacterial infections and is frequently used as a marker of bacterial sepsis. However, elevated procalcitonin levels have been reported in some COVID-19 patients, despite the fact that it is generally thought to be a less reliable marker of viral infections [30,32,39,103]. The study by Todor et al. considered PCT a good predictor for mortality, having an area under the curve (AUC) of 0.852 and a hazard ratio (HR) of 4.414 in the multivariate analysis [36].

### 3.12. D-Dimers

A protein fragment called D-dimer is created when the body dissolves a blood clot. Patients with COVID-19 have been reported to have elevated D-dimer levels, which may be linked to a higher risk of thrombosis and worse clinical consequences [23].

Since the beginning of the pandemic, cases of elevated levels have been reported internationally and in Romanian patients, with a tendency to be even higher in elders, patients with a history of heart conditions, or severe cases [14,21,24,25,27,31,33,36,39,40,43,45,62,79,89].

Moreover, the study by Dumache et al. proposed this analyte as a prognostic marker in monitoring the evolution of SARS-CoV-2-infected patients, considering it correlated extremely well with viral load [104]. Another relationship was established by Tudoran et al., indicating that D-dimer levels correlated inversely with lower levels of estimated systolic pulmonary artery pressure, which is used as an indicator of pulmonary hypertension [73].

Timpau et al. concluded that its use as an independent risk factor for ICU admission and death was relevant, with a cutoff of 2.05 mg/L (Se. = 65.7%, Sp. = 70.8%) [60]. Other studies corroborated this idea later in the pandemic [26,36], but some authors have affirmed that they appeared to be more frequently associated with PIMS than with SARS-CoV-2 infection [41,63,83].

In regard to treatment, Radulescu et al. observed an association between improved levels of D-dimers and the use of Tocilizumab [70].

### 3.13. Fibrinogen and Other Clotting Markers

Fibrinogen is a protein that is involved in blood clotting and is often elevated in response to inflammation or tissue damage. Elevated fibrinogen levels have been reported in COVID-19 patients and may be associated with an increased risk of thrombosis and poor clinical outcomes [21,23,25,27,29,33,36,39,40,43,48,72,89]. Furthermore, Mihai et al. found that elevated fibrinogen levels were associated with PIMS, likely due to the systemic inflammatory response that is characteristic of the condition [41].

One study by Davidescu et al. claimed that fibrinogen was further increased in elderly patients with new neurological manifestations at presentation. They also observed an increase in the activated partial thromboplastin time (aPTT) but not in the prothrombin time (PT) [62]. This aPTT and PT observation was discussed by other authors as well [21,37].

Fibrinogen was also associated with the levels of estimated systolic pulmonary artery pressure in COVID-19 patients, showing an inverse relationship and was even higher in patients with altered diastolic and left ventricular systolic functions according to research by Tudoran and his teams [45,73,105]. In Pliut and his team’s study, fibrinogen correlated well with the NYHA classification in COVID-19 patients [33].

Significantly lower survival rates were observed for patients with increased international normalized ratios (INR), with a HR of 1.54, as found by Pal et al. [59].

Another identified component of the clinical condition of COVID-19 is the hyperactivation of the complement and coagulation systems through the complement system, which is essential to this inflammatory response. In fact, patients with severe COVID-19 exhibit substantial complement activation in their sera, skin, and lungs; nevertheless, after receiving treatment with complement inhibitors, all of these patients healed without experiencing any negative side effects [106,107]. A decreased complement C3 level and the presence of schistocytes were found in the study by Pinte et al.; however, the patient suffered from a gene mutation in the complement factor I gene [80].

### 3.14. Blood Urea Nitrogen (BUN) and Other Renal Markers

Renal function is one of the most important actions the body is capable of, as it is, along with bile excretion, the way to eliminate toxic metabolites. As such, it is no wonder why many authors have reported on different markers that characterize the urinary tract [108]. BUN is a measure of the amount of nitrogen in the blood that comes from urea, which is a waste product of protein metabolism that is normally excreted by the kidneys. Elevated BUN levels may indicate impaired kidney function, which is a common complication of severe COVID-19 disease. Many authors have reported high levels in data from Romania and suggested a link with the severity of the infection, as well as a possible marker for severity [26,32,33,34,40,62].

Some authors have reported other common renal markers such as creatinine and urea [30,40,59]. In the study by Totan et al., urea levels were similar across severity groups [56], while other authors have reported normal values in their patients as well [21,43]. Studies, such as that by Timpau et al., noticed increased levels of both creatinine and urea in non-survivors [60]. Other articles that noticed elevated levels of creatinine include [26,33,36,37,45]. By itself, creatinine was also statistically higher in patients with COVID-19 than in patients with PIMS [63,83].

On the subject of renal markers in specific populations, it was observed that the Roma population had a significantly higher level of creatinine than the general population of Romania [53].

### 3.15. Complete Blood Counts (CBC) and Other Blood Test Markers

Hematological factors that have been linked to COVID-19 infection and severity include platelets, white blood cell total count, lymphocytes, neutrophils (along with neutrophil-lymphocyte and platelet-lymphocyte ratios), and hemoglobin [109,110].

Although not specific, increased white blood cells (WBCs) have been reported since the start of the pandemic. Some patients have been observed to have an altered leucocyte formula, with the most common forms being neutrophilia and lymphopenia, while basophils and eosinophils remained about the same [56]. From the studied papers, neutrophilia and lymphopenia were observed in many Romanian patients [14,24,25,33,34,43,60,62,72,74,79,89,91]. This topic was further addressed by Citu et al. in 2022, and it was found that the neutrophil to leukocytes ratio (NLR), derived neutrophil to leukocytes ratio (dNLR), and monocyte to leukocytes ratio (MLR) were found to be good predictors of severity and mortality in patients with COVID-19, with a hazard ratio (HR) of 3.85, 6.4, and 3.05, respectively, on univariate Cox regression [111]. This is corroborated by Cocos et al., Hutanu et al., Todor et al. Restea et al. [26,29,36,48]. From this point of view, similar changes could be observed in pediatric cases [22,23,30,39,112].

On rare occasions, thrombocytopenia might be observed [22,35,38,64,80,112]. Even rarer were the cases of thrombocytosis [39]. Another hematological problem, which is mostly linked to iron problems, is that anemia can also be present [23,36,37,38,39,40,112]. In the study by Uta et al., anemia was observed in pregnant women with SARS-CoV-2 infection, and they were urged toward supplementation therapies [88].

Erythrocyte sedimentation rate (ESR) is a non-specific marker showing a proinflammatory state. Although it is less specific than PCT or CRP, it is one of the most commonly used and accessible tests. Elevated levels have been observed in most cases of patients from Romania suffering from COVID-19 [21,23,33,36,39,40,48,54,89]. In Melit et al.’s study, there was a statistically significant difference between pediatric patients with COVID and those suffering from PIMS, with the latter being higher [83].

### 3.16. Microbiological Cultures and Assessments

The severity of respiratory viral infections can often be exacerbated by bacterial coinfections. Staphylococcus aureus, Streptococcus pneumoniae, and Haemophilus influenzae are some of the most prevalent pathogens observed in new COVID-19 cases. Pseudomonas aeruginosa, Klebsiella spp., and S. aureus can also be prevalent pathogens in patients with protracted hospitalization. Although most COVID-19 patients admitted to hospitals do not require antibiotics or bacterial infection diagnostic tests, doctors should keep an eye out for patients with coinfections and nosocomial bacterial infections [113,114].

As such, many authors have reported on bacterial co- or supra-infections in around half of COVID-19 cases from the ICU (50.42%, 95% CI = 43.86–56.97) [32], noticing cases of carbapenem-resistant Klebsiella pneumoniae in ICU patients with a heightened rate of mortality (30–70%) [115]. Other important bacterial species were Acinetobacter baumannii, Pseudomonas aeruginosa, Negative Coagulase cocci, Enterococcus spp., and Staphylococcus aureus [32].

The authors of another study also found that COVID-19 patients with commensal flora respiratory co-infections had a significantly higher proportion of patients with elevated WBC, while patients with fungal co-infections had significantly higher lymphocyte counts and transaminase levels [40]. When considering patients with type 2 diabetes, Gradisteanu et al. observed a decreased alpha diversity of the microbiome compared to controls, as measured by the fact that the Shannon index, lower fecal butyrate levels, and IL-8 levels were positively associated with Enterobacteriaceae, Sutterella, Bacteroidaceae, Clostridiaceae, and Parasutterela, while IL-17 expression was positively correlated with Sutterellaceae, Alistipes, and Enterobacteriaceae [49]. According to Susan et al., MDR bacteria were involved in 73.17% of COVID-19 patients with bacterial superinfections. Klebsiella pneumoniae, Enterococcus spp., and MRSA were the most common MDR bacteria identified in late infections after hospitalization in 20.43%, 4.30%, and 4.30% of all infections, respectively [116].

### 3.17. Other Markers: Thyroid, Antibodies and Ablumin

An early study by Badiu et al. looked into patients with a history of hepatitis C virus infection and thyroid disease who contracted COVID-19 and noticed an increase in antithyroid antibodies, regardless of their baseline antibody levels. While patients with autoimmune thyroiditis had a significant increase in anti-thyroid peroxidase antibodies (ATPO) and anti-thyroglobulin antibodies (anti-Tg) levels at a 1-month follow-up after contracting the respiratory infection, patients with euthyroidism had a significant decrease in thyroid-stimulating hormone (TSH), free triiodothyronine (fT3), and free thyroxine (fT4) levels. As such, it was concluded that thyroid involvement and its markers should not be overlooked [117].

Antibodies have been used in parallel with PCR testing for the detection of SARS-CoV-2 and are implemented at a later stage. According to some research, the reliability of this diagnostic test is similar to that of PCR; however, it does have one major setback. Antibodies are only available after the patient’s exposure to the virus and after a few days of illness. The majority of patients have SARS-CoV-2-specific IgG, IgM, and IgA antibodies as well as viral RNA [118,119].

This type of analysis has proven very useful in monitoring healthcare workers according to Manolea et al. (OR = 13.75 in comparison to patients) [120]. Furthermore, IgG antibodies can be found for up to 6 weeks [89] or longer [21,121]. After the development of vaccines against SARS-CoV-2, antibody titers proved a very helpful method of verifying how efficient they were. Studies by Maruntelu et al. and Olariu et al. from 2021 did observe post-vaccination IgG titer presence in all studied patients, yet immunization decreases over time, with the conclusion that previously infected individuals had higher antibody levels after vaccination than those who were not previously infected [122,123]. Regarding vaccine effectiveness and antibody titers, Ionita and his team observed good responses from both the BNT162b2 and the mRNA-1273 COVID-19 vaccines and noticed a humoral response after the 2-dose vaccination, resulting in a protective antibody level of 90% and 97%, respectively, in patients with hemodyalisis [124]. According to Korodi et al., a gradual drop in IgG levels was noticed, with the infection-naive cohort having statistically significant lower median values even 7–8 months after vaccination compared to the previously infected cohort (0.7 AU/mL versus 1.29 AU/mL, respectively) [125].

There is a downside to using IgG/IgM antibodies in regard to the significant variability in sensitivity of tests from different companies, yet they all provide good, high specificity [126]. Following BNT162b2 vaccination, it was suggested that adiponectin would be a better marker for predicting the immune responses to the vaccine [55].

The results of the study conducted by Vata et al. showed that the early detection of IgA was more frequent in patients who later developed severe forms of the disease [127].

On occasion, hypoalbuminemia was reported in COVID-19 patients, but it correlated better with PIMS being further decreased in the pediatric patients suffering from this post-vial condition [23,41,83].

## 4. Discussions

The COVID-19 pandemic, caused by the novel coronavirus SARS-CoV-2, has posed unprecedented challenges to global public health systems, economies, and societies. Every country has experienced the pandemic’s impact differently, with varying rates of infection, healthcare system strain, and socioeconomic consequences. This article focuses on understanding the impact of COVID-19 on patients in Romania, highlighting its research regarding laboratory markers and their role in the infection. Laboratory markers are crucial tools used by healthcare professionals to gain insights into the disease’s severity, progression, and potential complications. The major laboratory markers that were studied by Romanian authors are displayed in Table 1.

In addition, this article aimed to identify gaps in this research and provide encouragement for Romanian research to focus on novel and detailed aspects of the pandemic. For instance, as Romania grapples with the aftermath of the COVID-19 pandemic, understanding the long-term effects of the disease is of paramount importance. PIMS is a rare but serious condition that predominantly affects children and adolescents who have had a recent COVID-19 infection. It shares similarities with Kawasaki disease and Toxic Shock Syndrome, often presenting with fever, rash, gastrointestinal symptoms, and cardiac inflammation [128,129]. There were a number of papers with this focus, in which it was observed that CRP, CK-MB, troponins, bilirubin, D-dimers, fibrinogen, creatinine, ESR, and albumin had modified levels [23,41,62,63,83]. These findings are in accordance with international studies [130,131]. Contrary to these findings in pediatric patients, data on the adult version of PIMS, called long-COVID or post-acute COVID, were even more limited, with all studies focusing on the cardiovascular aspects in such patients [45,46,51]. As such, this is one area where more laboratory markers should be covered, as, internationally, a team from Brazil has outlined the hematological alterations in long COVID [132].

Other markers that lack research in Romanian studies include, but are not limited to, thyroid markers, adiponectin, specialized cell populations such as natural killers, and complement levels. Recent studies have hinted at the possibility of SARS-CoV-2 affecting mitochondrial function within host cells. Investigating this potential interaction is paramount, as mitochondria play a central role in cellular energy production, apoptosis, and immune responses. Unraveling the mechanistic intricacies of mitochondrial infection could elucidate the diverse clinical manifestations of COVID-19 and inspire innovative therapeutic strategies [133]. At the moment, no research in this area has been carried out on patients from Romania, prompting us to encourage the discussion of this subject.

The quest for effective antiviral treatments for COVID-19 remains a global priority. Research into novel therapeutic approaches, including repurposing existing drugs, developing antiviral agents, and harnessing innovative immunomodulatory strategies, holds immense promise. Yet, few studies in the selected lot contained explicit laboratory marker data in patients treated with antiviral drugs [67,98]. While both studies looked into the effects of Remdesivir, one followed its efficacy in children, where the authors noticed elevated transaminase, CRP, LDH, prothrombin time, anemia, and lymphopenia according to COVID severity at the beginning of the treatment, concluding that transaminase levels and prothrombin time were elevated after the Remdesivir intake, which were considered secondary effects of the treatment [98].

The other was a comparative study in adults, in which Remdesivir was paired with either Tocilizumab or Anakinra. Here, the authors concluded that CRP, FER, and LDH had a favorable evolution more quickly in patients treated with Tocilizumab than in patients treated with Anakinra, which had a slower development. [67]. Furthermore, Favipiravir was only studied in a case series paper [134]. Overall, the relationship between antiviral drugs and laboratory markers was scarcely documented in Romanian patients. With that in mind, the need for more in-depth studies regarding COVID-19 therapies in general, and antiviral treatment, specifically, needs to be continuously emphasized.

Another important aspect to keep in mind is the Roma population, which is numerous in Romania. The findings show that community health partnerships between minority group organizations and medical professionals can lessen the pandemic’s disproportionate effects on Roma. The COVID-19 pandemic has magnified the systemic inequalities that Roma communities face, highlighting the imperativeness of transformative change [135,136]. By addressing these disparities, fostering inclusivity, and recognizing the diversity of experiences within the Roma populations, we can work toward a more equitable pandemic response and lay the groundwork for a more just and resilient society in the future.

## 5. Conclusions

The impact of COVID-19 on patients in Romania encompasses multiple dimensions, from public health and epidemiology to healthcare system strain, vulnerable populations, variants, socioeconomic consequences, international collaboration, and policy implications. By focusing on patients in Romania, researchers and policymakers can gain a comprehensive understanding of the pandemic’s effects within this country. This knowledge is essential for devising targeted interventions, informing policy decisions, and contributing to the global efforts aimed at overcoming the challenges posed by COVID-19.

COVID-19 has rapidly spread globally and has significantly impacted Romania. Biomarkers play a crucial role in understanding and managing the disease. PCR testing is still considered the gold standard, with pooling methods able to reduce costs and the number of tests needed for large-scale testing while maintaining high accuracy. Some biomarkers, such as CRP, LDH, transaminases, cardiac and iron markers, etc., have been extensively researched in Romanian patients, while others are still disputed (albumin, CK, PCT, thyroid hormones, etc.). CRP, IL-6, LDH, FER, fibrinogen, creatinine, and vitamin D (to a lesser extent) levels have been associated with increased severity and risk of ICU admission and death and proved good predictors, even in antiviral treatment monitorization. Cardiac markers and D-dimers proved to be good predictors as well, but their role tends to be more important in children with PIMS. Regarding cholesterol, HDL generally proved a better predictor than LDL. Elevated BUN levels are linked to severity and can be considered a potential marker for severity.

Regarding hematological issues in SARS-CoV-2 infections, neutrophilia and lymphopenia were common forms, prompting the use of their ratio (NLR) as a potentially useful marker for severity. ESR elevation is present in virtually all patients, but it is too non-specific to be a useful marker. IgG, IgM, and IgA antibodies proved to be reliable analyses; however, their lifespan in vaccinated persons is considerably shorter than in infection-naïve individuals. PCT is generally a marker of bacterial infections and should be taken into consideration when dealing with co- and supra-infections, of which Klebsiella pneumoniae, Acinetobacter baumannii, Pseudomonas aeruginosa, Enterococcus spp., and Staphylococcus aureus were among the top etiological agents.

As such, it is becoming increasingly evident that developing and utilizing specialized laboratory markers and more detailed analyses for Romanian patients affected by this disease is crucial. The suggested topics in which biomarkers in COVID-19 patients should be studied are long-COVID or post-acute COVID, immune response variability, variant characterization, novel therapeutic approaches, mental health impact, and the long-term effects of children who were infected.

Overall, there is also a need to address health disparities, vulnerable populations, and minority issues in regard to the pandemic, with one such population being the Roma, a historically marginalized people. As we navigate the challenges of the pandemic and its aftermath, our success will be measured by our ability to ensure that no one is left behind, regardless of their ethnicity or background.

## Figures and Tables

**Table 1:** Laboratory markers and assessments that were frequently observed in Romanian studies.

Assessment	General Role	Most Frequent Modification	Studied Populations	Predictive Ability (Ref.)	Clinical Associations (Ref.)
RT-PCR	Early detection; diagnosis	Positive	General	-	-
Antigen levels—IgM, IgG	Diagnosis; post-vaccination monitoring	Elevated	General	-	-
CRP	Disease severity assessment; monitoring progression; therapeutic guidance	Elevated	Adults, children, infants, elders, Roma	Yes, even more so in Roma [53,54,58,59,60,61,62,70]	Diabetes [48,94] Antiphospholipid syndrome [47] Hypertension [27] Cardiac dysfunction [46,73,93] Overweight/metabolic syndrome [27,54,93] Lung involvement [50,57] PIMS [83] Chronic Spontaneous Urticaria Exacerbation [42] Pneumothorax/pneumomediastinum [31,72] Liver failure [44] Multiple organ dysfunction [60]
LDH	Disease severity assessment; monitoring progression; therapeutic guidance	Elevated	General	Yes [57,58,61]	Lung involvement [57] Chronic Spontaneous Urticaria Exacerbation [42]
FER	Disease severity assessment	Elevated	Adults, children, elders	Yes [58,60,70]	Pneumothorax/pneumomediastinum [31,72]
HDL	Disease severity assessment	Elevated	Adults, children, elders	Yes [46,58,93]	-
Vitamin D	Disease severity assessment	Decreased	Adults, children, elders	Yes [57,58,102,103] No [55]	More comorbidities [29,101] Lung involvement [57]
PCT	Disease severity assessment; evaluation of complications	Elevated	Adults	Yes [36]	Diabetes [48] Co-/supra-infections [40,115,116]
D-dimers	Disease severity assessment; monitoring progression; evaluation of complications	Elevated	Adults, elders	Yes [26,36,60,105]	Hypertension, overweight [27] Cardiac dysfunction [73] Liver failure [44] PIMS [83] Pneumothorax/pneumomediastinum [31,72] Liver failure [44] Multiple organ dysfunction [60]
BUN	Disease severity assessment	Elevated	Adults	-	-
Neutrophils	Disease severity assessment; monitoring progression	Increased	General	-	-
Lymphocytes	Disease severity assessment; monitoring progression	Decreased	General	-	-
Platelets	Disease severity assessment; monitoring progression	Decreased	General	-	-
NLR	Disease severity assessment; monitoring progression; therapeutic guidance	Increased	General	Yes [26,29,36,48,111]	Lung involvement [50]
ESR	Evaluation of complications	Increased	Children	-	-
Cytokines—IL-1, IL-6, IL-8, IL-1β, IFN-γ, TNF-α, TGF- β	Monitoring progression; therapeutic guidance	Elevated	Adults, children, elders, Roma, pregnant and lactating women	Yes; IL-6, especially in Roma [36,53,59,60]	IL-6: hypertension, overweight [27,54]; lung involvement [50,72]; cardiac dysfunction [73]; multiple organ dysfunction [74]; liver failure [44] IL1β, IL-8: bacterial infection [49]
Tn’s	Monitoring progression	Elevated	Adults	Yes [33] No [21,86]	Cardiac dysfunction [33]
BNP	Monitoring progression	Elevated	Adults, children	Yes [33] No [21,86]	Cardiac dysfunction [33]
AST and ALT	Therapeutic guidance	Elevated	Remdesivir side-effects in children	-	
Fibrinogen	Evaluation of complications	Elevated	Adults, children	Yes [33,41,62] No [29,61,79]	PIMS [41] Cardiac dysfunction [33]
Creatinine	Evaluation of complications	Elevated	Children, elders, Roma	Yes [53,59,62,83]	PIMS [83] Renal dysfunction [41,59]
Albumin	Evaluation of complications	Decreased	Children	-	-

## Data Availability

Data regarding the selected materials are available upon request from the correspondence author.
